# Implementing a hip arthroplasty program in Greenland, the autonomous territory of Denmark: challenges and outcomes

**DOI:** 10.1007/s00264-026-07034-4

**Published:** 2026-09-15

**Authors:** Mikolaj Bartosik, Najannguaq Jørgensen, Reinhard Stoewe

**Affiliations:** 1 Department of Orthopaedics and Trauma Surgery, Ludwig-Maximilians-Universität München, Munich, Germany; 2 Department of Orthopedic Surgery, Aalborg University Hospital, Aalborg, Denmark; 3 Department of Orthopedic Surgery, Queen Ingrid Hospital, Nuuk, Greenland

**Keywords:** Hip arthroplasty, Greenland, Hip replacement program, Femoral fracture

## Abstract

**Background and purpose:**

Greenland is undergoing rapid societal change, while its healthcare system faces substantial geographic and structural challenges. In January 2024, a routine program was established for elective total hip arthroplasty (THA) and hip replacements following femoral neck fractures.

**Patients and methods:**

All patients receiving hip replacement from January 2024 to January 2026 were analyzed retrospectively. Demographic parameters, complications, revision rates, one month mortality were evaluated and compared primarily with register data from Denmark, Greenland’s parent country.

**Results:**

A total of 123 patients (129 procedures) were assessed. Elective THA patients were younger (60 vs 68 years), had a higher body mass index (BMI) (29.9 vs 27.6), similar American Society of Anesthesiologists (ASA) scores, longer operative times (110 vs 92 min), lower blood loss (ΔHb -2.1 vs -2.3 g/dL), longer length of stay (discharge post-OP day 4.2 vs 1) and substantially higher complication rates than Danish controls. Hip fracture patients were younger (74 vs 79 years), had a lower BMI (23.9 vs 26), had a similar ASA distribution, lower blood loss (ΔHb -1.3 vs -3.4 g/dL), a similar post-OP discharge day (7.9 vs 7), and also numerically higher complication rates than Danish controls.

**Conclusion:**

Hip arthroplasty outcomes in Greenland were less favorable than in Denmark, likely due to various systemic and patient-related factors. Continuous efforts are required to optimize patient safety and clinical outcomes in this setting.

## Introduction

Greenland is transitioning rapidly from a traditional society to a highly developed one [1]. Its 56,000 residents are distributed over a vast territory, with one third living in the capital Nuuk and the remainder spread across 16 smaller towns and 55 remote settlements [2]. Geographic isolation, extreme climatic conditions, and transport logistics, which account for 12% of health expenses, complicate healthcare delivery.

Compared to Denmark, Greenland faces distinct baseline public health challenges, including higher rates of infectious diseases, smoking, and diabetes, as well as higher suicide rates and a lower life expectancy (Table 1).

**Table 1:** Health characteristics of Greenland and Denmark

	Greenland	Denmark	
Population (2025)	56,583	6,028,459	[2, 3]
Life expectancy (2025)	74.0	80.8	[2–4]
Number of inhabitants/medical doctor (2025)	471	207	[2, 3]
Number of inhabitants/dentist (2025)	2021	1443	[2, 3]
Percentage of obesity (BMI ≥ 30) (2025)	28.2	13	[1, 2, 5]
Percentage smokers (2025)	75	10.5	[2, 5, 6]
Percentage HIV (2025)	0.2	0.005	[2, 5]
Prevalence tuberculosis (2006–2022)	149	2	[7]
Percentage diabetes (2017)	10	8.3	[1, 2, 5]
Number of THA/100000 inhabitants (2024)	68	234	[8]
Suicides/100000 inhabitants (2018)	81	11.2	[2, 9]
Percentage of population attained tertiary education (2025)	16.0	45.1	[2, 3]

This table presents synthesized population data for Greenland and Denmark, extracted from the literature. Due to group heterogeneity and limited data availability, representative subgroups were occasionally utilized or a cross-year projection was conducted

Prior to 2024, elective hip arthroplasty was severely restricted, creating substantial waiting lists. In January 2024, a routine hip replacement program was launched at Queen Ingrid Hospital in Nuuk, the sole health facility in Greenland capable of performing these procedures.

This study evaluates the outcomes, complications, and structural limitations of the initial 25-month cohort, hypothesizing that geographic isolation and resource constraints negatively impact surgical outcomes compared to Danish national benchmarks.

## Materials and methods

### Study design and data collection

This retrospective cohort study included all consecutive patients who underwent hip arthroplasty at Queen Ingrid Hospital between January 1, 2024, and January 31, 2026. Patients were divided into two groups: (1) elective total hip arthroplasty and (2) hip arthroplasty following femoral neck fracture. Protocols were approved by the Greenlandic Ethical Scientific Committee (2024–4357) and the Greenlandic Data Protection Agency. Data were prospectively recorded, including demographics (age, sex, BMI, ASA score), surgical metrics, technical difficulties, complications, revisions, and one month mortality. Postoperative follow-up averaged 18 months (range 6–28 months) via clinical visits, radiographs, telephone calls, and integrated electronic medical records. Because Greenland is part of Denmark, demographic and surgery-specific data were compared, when possible, with the Danish registry data and otherwise with studies involving similar hip replacement programs in developed countries.

In Greenland, clinicians do not have access to the Danish hip arthroplasty registry, and applications to report Greenland’s results to the Danish hip arthroplasty registry were repeatedly rejected. Consequently, comparisons could not be performed on a patient-by-patient basis but rely on published aggregate data specific to each outcome measure. For the elective cohort, dislocation, PJI, and revision rates were benchmarked against Danish Hip Arthroplasty Register-based studies [10–12], and age, BMI, and discharge timing against a Danish fast-track cohort [13]. For the fracture cohort, age, BMI, and ASA distribution were derived from a national-registry-based study [14], time-to-surgery and mortality from the Danish Fracture Database [15], discharge timing from a national mortality register study [16], and dislocation, PJI, and reoperation rates from further Danish registry-based cohorts [17, 18]. Transfusion rates and ASA distributions were compared with international rather than Danish cohorts [19, 20]. As only published summary statistics were available, no adjustment for confounding or direct statistical matching could be performed.

We applied Infectious Diseases Society of America (IDSA) criteria [21], which were adapted to local conditions. This modification did not affect the validity of the diagnosis or the results of the treatment.

### Operation and implants

Preoperative planning was conducted digitally using TraumaCad® software. All surgeries were performed via a direct lateral Hardinge approach in the lateral decubitus position. The patients received perioperative antibiotic prophylaxis for one day, as well as intravenous tranexamic acid at a dose of 1 g before surgery and 1 g three hours after the first dose. All implants, supplied by DePuy Synthes, were uncemented due to local economic constraints precluding cemented options. THA specifications followed Danish guidelines using 32 mm metal heads on polyethylene liners. Supply chain lead times for replenishment were approximately two weeks.

### Statistics

GraphPad Prism 10.4.1 (GraphPad Software, San Diego, CA, USA) was used for statistical analysis. Results are presented as mean ± standard deviation (SD), min, and max value. A descriptive comparison was conducted between our data and the data from the Danish and international registries based on the missing raw data.

## Results

### Cohort overview

A total of 123 patients (129 hips) were included.

#### Elective cohort (Table 2)

Eighty-three patients (88 hips; THA 87, distal hip hemiarthroplasty (DHA) 1). Indication: osteoarthritis 68, childhood hip disorder sequelae nine, caput necrosis nine, rheumatoid arthritis two.

**Table 2:** Patients receiving an elective hip replacement

Elective patients Greenland (*n* = 88)						
Parameter	*n*	%	Mean	SD	Min	Max
Demographics						
Women/men	33/55	38%/62%				
Age (years)	-	-	60	10.6	25	80
BMI (kg/m^2^)	-	-	29.9	5.1	20	42
ASA			2.0	0.5	1	3
ASA 1	12	14%	-	-	-	-
ASA 2	67	76%	-	-	-	-
ASA 3	9	10%	-	-	-	-
ASA 4	0	0.0%	-	-	-	-
Operation						
Left/right	38/50	43%/57%	-	-	-	-
THA/DHA	87/1	99%/1%	-	-	-	-
Operative time (min)	-	-	110	20	75	170
Perioperative blood loss (Δ Hb (g/dL))	-	-	−2.1	0.8	−0.8	−4.2
Received blood transfusion n =	0	0.0%	-	-	-	-
Discharge day post-OP	-	-	4.2	2.8	1	21
Complications						
Dislocation	5	5.7%	-	-	-	-
Deep surgical site infection	3	3.4%	-	-	-	-
Others leading to revision surgery	1	1.1%	-	-	-	-
Revisions	8	9.1%	-	-	-	-
Death	0	0.0%	-	-	-	-

#### Fracture cohort (Table 3)

Forty patients (41 hips; THA 17, DHA 24). Indication: displaced femoral neck fractures (Garden III: 26, Garden IV: 15).

**Table 3:** Patients receiving a hip replacement due to femoral neck fracture

Hip fracture patients Greenland (*n* = 41)						
Parameter	*n*	%	Mean	SD	Min	Max
Demographics						
Women/men	19/22	46%/54%				
Age (years)	-	-	74	8.6	54	87
BMI (kg/m^2^)	-	-	23.9	4.9	17	36
ASA			2.5	0.6	2	4
ASA 1	0	0.0%	-	-	-	-
ASA 2	21	51%	-	-	-	-
ASA 3	18	44%	-	-	-	-
ASA 4	2	5%	-	-	-	-
Operation						
Surgical delay trauma to surgery (days)			48.8	181	1	1100
Hospital delay admission to surgery (days)			2.5	1.3	1	7
Left/right	25/16	61%/39%				
THA/DHA	17/24	41%/59%				
Operative time (min)	-	-	87	22	48	170
Perioperative blood loss (Δ Hb (g/dL)	-	-	−1.3	1.0	0	−3.4
Received blood transfusion	5	12%				
Discharge day post-OP	-	-	7.9	9.3	2	61
Complications						
Dislocation	4	9.8%	-	-	-	-
Deep surgical site infection	1	2.4%	-	-	-	-
Others leading to revision surgery	0	0.0%	-	-	-	-
Revisions	4	9.8%	-	-	-	-
Death	3	7.3%	-	-	-	-

### Minor surgical & non-surgical complications

Across all 129 procedures, nine minor surgical complications occurred: one acetabular undercoverage, one minor medial wall damage, three stem malalignments (> 3° varus/valgus), and four intraoperative undislocated trochanteric fractures treated with cerclage. Seven non-surgical complications occurred: two acute renal failures, one urinary retention, one ileus, one acute psychosis, one severe gastritis, and one postoperative malaria attack, all of which resolved with targeted medical management.

### Patients and complications – elective THA

Compared to international reference cohorts, Greenlandic elective patients were younger (mean 60 years, SD 10.6, range 25 to 80) vs. (mean 68 years, SD 15) [19], predominantly male (62% vs. 43% male internationally) [19] and had a higher BMI (mean 29.9, SD 5.1, range 20 to 42) than in a comparable Danish group (mean 27.6, SD 5) [13]. No patient needed a postoperative blood transfusion, aligning with international rates (< 1%) [20]. However, postoperative hospital stay was longer (mean 4.2 d (SD 2.8, range 1 to 21) vs. 1 d in Denmark) [13]. Severe complication rates were higher than Danish registry data: dislocation (5.7% vs. 2.8%), PJI (3.5% vs. 0.86%), and revision (9.1% vs. 2.6%) [10–12].

### Patients and complications – proximal hip fracture cohort

Our fracture patients (mean age 74 years, SD 8.6, range 54 to 87) had a lower mean BMI (mean 23.9, SD 4.9, range 17 to 36) than a comparable group from Denmark (mean 26) [14] and a similar ASA distribution (mean 2.54, SD 0.6, range 2 to 4 vs. mean 2.41 DK) [15]. For patients in Greenland, the time between the accident and surgery was long, with a median of 4.0 days (interquartile range 7.5, mean 48.8 days, SD 181, range 1 to 1,100). The delay from hospital admission to surgery alone was a mean of 2.5 days. The duration of surgery was a mean of 87 min (SD 22, range 48 to 170) with perioperative blood loss of ΔHb mean −1.3 g/dL (SD 1.0, range 0 to −3.4). The length of postoperative stay for this group of patients (mean 7.9 days, SD 9.3, range 2 to 61) was similar to DK (mean 7 days) [16].

Severe complication rates exceeded Danish registry data: dislocation (9.8% vs. 7.5%), PJI (2.4% vs. 1.9%), and revision (9.8% vs. 7.5%) [14, 17, 18]. However, one month mortality was lower (7.3% vs. 11%) [14].

### Management of major complications

#### Periprosthetic Joint Infection (PJI)

Four total PJI cases occurred across both cohorts. Three acute cases underwent Debridement, Antibiotics, and Implant Retention (DAIR) within 28 days; two achieved infection eradication, while one developed a chronic sinus tract (patient declined two-stage revision). The fourth case presented at day 35 and required transfer to Denmark for two-stage revision.

#### Dislocations and revisions

Nine patients experienced dislocation, with eight requiring revision due to irreducibility or recurrent instability. Remote geographical origin delayed acute presentation in several instances. Revisions were completed using constrained or dual-mobility components (six in Nuuk, two in Denmark). Two revision cases developed subsequent infection (one cleared via DAIR, one required Girdlestone resection). One elective THA underwent revision for acetabular component undercoverage (< 80%), thereafter proceeding well.

## Discussion

### Patients

The main aim of this study was to evaluate the outcome of a hip replacement program established in Greenland in 2024. Here, we examined the baseline health status of Greenlandic patients, the health system in Greenland, Queen Ingrid Hospital in Nuuk, operations, and outcomes, with a specific focus on postoperative complications. With the exception of postoperative mortality and intraoperative blood loss, the cohort’s overall clinical outcomes were inferior to those reported in comparable studies from Denmark or other developed countries.

Prior literature demonstrates that when implanting a hip prosthesis in more vulnerable patients in low-income settings or operating on patients with lower socioeconomic status, complication rates such as PJI (4.73% for THAs in a developing countries) [22], revision rates, and mortality [22, 23] increase. Other authors have already proposed better education, advancements in the operating room design and layout, and the introduction of large national registries to improve the situation [22]. When looking at the health data of our population in Greenland (Table 1) it seems plausible to assume a relatively unfavourable outcome of THA surgery compared to the outcome in Denmark [24]. Smoking increases the revision rate and risk of developing a postoperative PJI by a factor of 2 to 3 [25, 26]. Given that our patients are representative of the Greenlandic adult population, we also noted a high prevalence of smokers, which highlights a higher risk profile for these patients.

**Fig. 1:**
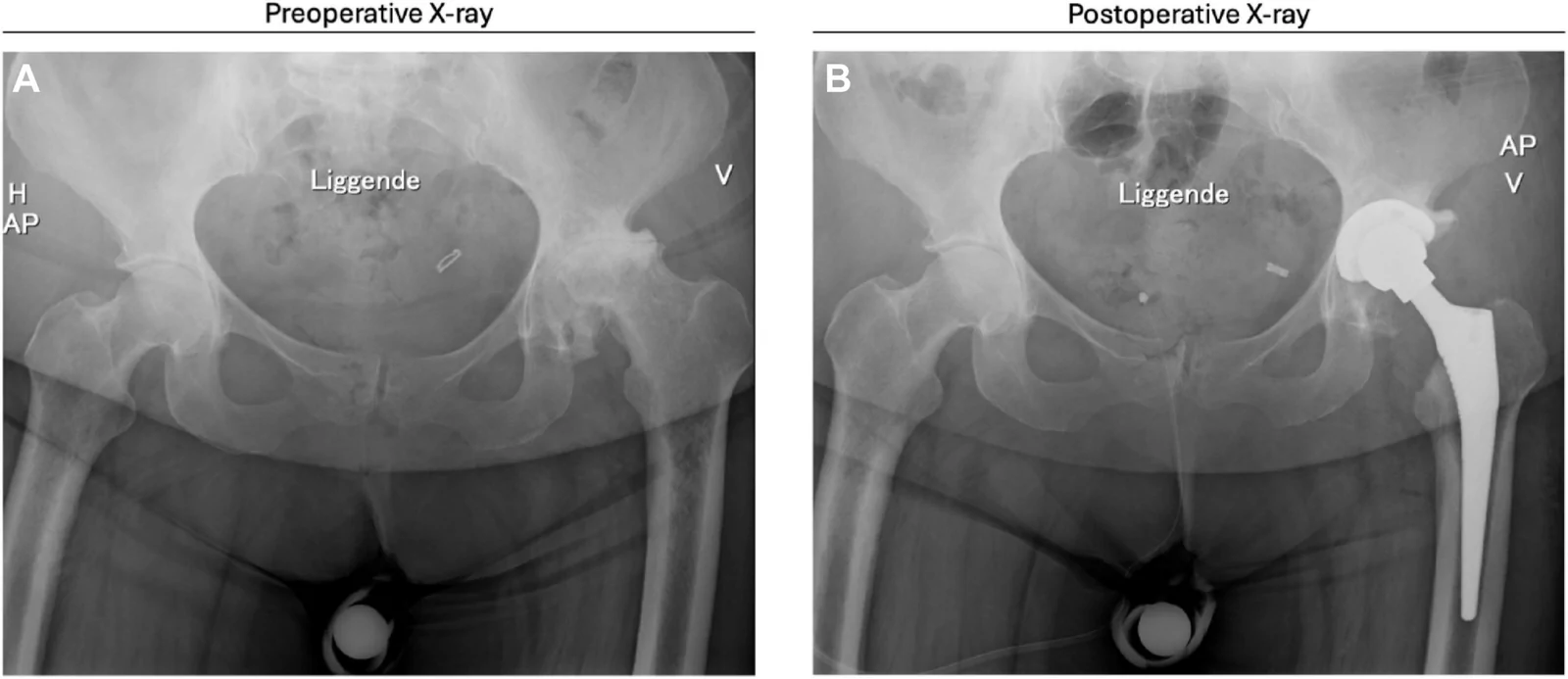
**A** and **B:** A typical Greenlandic patient receiving an elective THA

Figure 1A illustrates a preoperative x-ray of a typical Greenlandic patient awaiting elective hip replacement surgery. This patient lives in a remote settlement in Greenland. At the date of surgery in 2025, the patient was 49 years old. At the age of 39, she was diagnosed with osteoarthritis of the left hip. Three years later, she was put on the waiting list to receive a THA. During the seven years spent on the waiting list, the patient developed severe acetabular and femoral head erosion. Preoperatively, she had a leg length discrepancy of −19 mm. Figure 1B shows the post-OP x-ray. Postoperatively, her leg length discrepancy is + 3 mm.

While in other countries the discharge day after a THA depends mainly on the health and pain status of the patient, in Greenland, where many patients live in geographically isolated regions, it depends mainly on the flight schedule of Air Greenland and the weather conditions, which frequently lead to flight delays of several days. This leads to a later discharge day (Table 2 and 3).

### Health system

While all types of medical care and dental care are free of charge, it remains a challenge for the population to access the health care system due to significant economic and geographical hurdles.

Due to the distribution of the population in Greenland, long distances, and transport expenses, the operating surgeon often knows the patient to be operated only through basic demographic data and x-rays. The entire pre-hospital, intra-hospital, and post-hospital set-up reflects the unique geographic, economic, and cultural situation in Greenland, falling short of fully personalized patient care.

While mainland Denmark guarantees a maximum waiting time of one month for elective THA, no such guarantee exists in Greenland. The waiting time between being diagnosed with severe osteoarthritis of the hip and undergoing surgery varies between two and seven years. This also influences the surgery itself and the results (Fig. 1).

For 2025, the health expenditure per capita in Denmark was 6,584 Euro [27] while in Greenland it was 3,540 Euro [28]. It is known that a healthcare system with a universal health insurance for the whole population, as it exists in Greenland and Denmark, decreases disparities in treatment and outcomes between majority and minority groups. This has been demonstrated for both diagnoses covered in this article, elective THA and proximal femoral fractures [29, 30]. However, this policy does not automatically lead to the same good results for the entire population. Addressing these disparities requires ensuring that identical policies, staffing levels, and clinical guidelines are applied universally, regardless of whether a patient resides in Denmark or Greenland—even if geographic implementation requires greater financial resources.

### The Queen Ingrid Hospital in Nuuk

Approximately 50% of healthcare personnel at Queen Ingrid Hospital work on short-term (2–6 week) locum contracts. The lack of a stable surgical team impedes operational familiarity, increasing operative times and surgical friction, thereby leading to poorer outcomes for the patient [31] compared with Danish hospitals [32, 33].

Studies based on results from the Danish hip fracture registry state clearly that any delay in surgery for a patient with a hip fracture increases the morbidity and mortality [15]. These Danish practices align with international guidelines [34]. In our fracture group in Greenland, five out of 41 patients had a time to surgery of more than one month, up to two years. Even after excluding these five patients, our fracture patients experienced substantial delays (trauma-to-surgery mean 4.0 days, hospital admission to surgery mean 2.5 days). This delay increases patient morbidity and mortality [15, 34], and all attempts to optimize time to surgery should be made to improve postoperative outcome.

### Operation

The economic pressure to cut down on expenses and resources across all steps of the surgical process is higher in Greenland than in developed countries. Intraoperative workflow is often disrupted by operating room team unfamiliarity, distractions, and procedural compromises. These factors result in a prolonged operative time for hip prosthesis in Greenland (Table 2) compared to other places in developed countries [12] and lead to higher rates of perioperative complications [32, 35, 36]. Therefore, to improve the patient safety and the postoperative outcomes, every effort should be made to optimize surgical flow and reduce disruptions. Although a limited selection of implants occasionally increased operative time and difficulty, in our opinion, it did not significantly impact overall outcomes or complication rates. The longer operative time at our hospital did not negatively influence the perioperative blood loss (Table 2). Instead, blood loss in our cohort was lower than the blood loss in a comparable international control group.

Due to lack of economic resources and a vulnerable supply chain, compromises had to be made regarding implant availability, and therefore cemented hip prostheses are not available in Greenland. We recognize that this limitation conflicts with Danish guidelines, which recommend cemented hip prostheses for specific populations (e.g., elderly patients with limited mobility) [37]. When using uncemented stems, the primary increase in complications involves periprosthetic fractures [37], though we observed none in our cohort. We did not observe that this constraint had a major impact on our outcomes or complication rates.

### Outcome and complications

Tables 2,  3, and international comparisons demonstrate that patients undergoing hip prosthesis surgery in Greenland experienced less favourable outcomes and higher complication rates than those operated on in Denmark. Several contributing factors have been outlined in the sections on patient characteristics, healthcare system structure, hospital environment, and surgical logistics. The culmination of these factors explains the overall results.

### Limitations

This study is limited by its retrospective design, small sample size, and short follow-up duration (mean 18 months), which likely leads to an underreporting of late PJI or aseptic loosening. In remote settlements, where patients return post-discharge, access to laboratory services, x-ray facilities, and specially trained staff to evaluate potential postoperative complications is severely limited. Furthermore, logistical isolation, cultural differences, and communication gaps may lead to underreporting of post-discharge complications, particularly outside Nuuk. Potential underreporting of postoperative complications further reinforces the observed disparity between hip replacement outcomes in Greenland and mainland Denmark, thereby supporting our conclusion.

To our knowledge, this is the first study to evaluate disparities in the surgical management of hip prosthesis patients between Greenland and Denmark. A key limitation of this study is the small sample size of patients. While the small sample size is a limitation, we do not view this as a reason to exclude Greenlandic patients from orthopedic literature or Danish national registries, as exclusion could further impair resource allocation and guideline implementation.

## Conclusion

Establishing a routine arthroplasty program in Nuuk has provided essential surgical access to an underserved population. However, outcomes and complication rates remain inferior compared to mainland Denmark. Overcoming these disparities requires infrastructure investment, standardized surgical pathways, enhanced emergency transport networks, staff stabilization, and formal integration of Greenlandic data into Danish national quality registries [8].

## Data Availability

All data supporting the findings of this study are available within the paper. Herewith, the authors confirm that all data and materials, used for this study, are available on request.

## Abbreviations

*ASA*: American Society of Anesthesiologists
*BMI*: Body mass index
*D*: Day(s)
*DAIR*: Debridement antibiotics implant retaining
*DHA*: Distal hip hemiarthroplasty
*DK*: Denmark
*GL*: Greenland
*Hb*: Hemoglobin
*IDSA*: Infectious diseases society of America
*OP*: Operation
*PJI*: Periprosthetic joint infection
*SD*: Standard deviation
*THA*: Total hip arthroplasty
